# Blinatumomab in Children and Adolescents with Relapsed/Refractory B Cell Precursor Acute Lymphoblastic Leukemia: A Real-Life Multicenter Retrospective Study in Seven AIEOP (Associazione Italiana di Ematologia e Oncologia Pediatrica) Centers

**DOI:** 10.3390/cancers14020426

**Published:** 2022-01-15

**Authors:** Giuliana Beneduce, Antonia De Matteo, Pio Stellato, Anna M. Testi, Nicoletta Bertorello, Antonella Colombini, Maria C. Putti, Carmelo Rizzari, Simone Cesaro, Monica Cellini, Elena Barisone, Fara Petruzziello, Giuseppe Menna, Rosanna Parasole

**Affiliations:** 1 Department of Pediatric Hemato-Oncology, AORN Santobono-Pausilipon, Hospital, 80123 Naples, Italy; g.beneduce@santobonopausilipon.it (G.B.); a.dematteo@santobonopausilipon.it (A.D.M.); fara.petruzziello@santobonopausilipon.it (F.P.); g.menna@santobonopausilipon.it (G.M.); r.parasole@santobonopausilipon.it (R.P.); 2 Division of Hematology, Department of Translational and Precision Medicine, ‘Sapienza’ University, 00161 Rome, Italy; testi@bce.uniroma1.it; 3 Stem Cell Transplantation and Cell Therapy Division, Department of Pediatric Onco-Hematology, A.O.U. Città della Salute e della Scienza—Regina Margherita Children’s Hospital, 10126 Turin, Italy; nbertorello@cittadellasalute.to.it (N.B.); elena.barisone@unito.it (E.B.); 4 Pediatric Hematology-Oncology Unit, Department of Pediatrics, University of Milano-Bicocca, MBBM Foundation c/o ASST Monza, 20900 Milan, Italy; acolombini@fondazionembbm.it (A.C.); c.rizzari@asst-monza.it (C.R.); 5 Department of Woman and Child Health, Clinic of Pediatric Haematology-Oncology, University of Padova, 35128 Padova, Italy; mariacaterina.putti@unipd.it; 6 Pediatric Hematology Oncology Unit, Department of Mother and Child, Azienda Ospedaliera Universitaria Integrata, 37129 Verona, Italy; simone.cesaro@aovr.veneto.it; 7 Pediatric Hematology Oncology Unit, Department of Mother and Child, Azienda Ospedaliero Universitaria Policlinico di Modena, 41125 Modena, Italy; cellini.monica@aou.mo.it

**Keywords:** targeted therapy, acute lymphoblastic leukemia, pediatric, relapse, toxicity, real-life experience

## Abstract

**Simple Summary:**

Blinatumomab, a bispecific T-cell engager, binding T-cell CD3 and B-cell CD19 antigens, has remarkably enlarged the treatment options for patients with relapsed/refractory B-cell precursor acute lymphoblastic leukemia (r/r BCP-ALL). The aim of our study was to retrospectively assess the safety and efficacy profile of blinatumomab in 39 r/r ALL children treated in seven AIEOP centers in a compassionate or off-label program. This report is among the largest multicentric real-life retrospective analyses on blinatumomab in pediatric r/r BCP ALL. Blinatumomab showed a tolerable safety profile (34.8% of adverse events ≥grade 3, no cytokine release syndrome and no associated toxic deaths) and proved to be very effective (complete remission rate 46% in the 13 patients with ≥5% blasts and 81% PCR/FC MRD negativity in the 26 patients with <5% blasts) in this group of patients affected by r/r BCP-ALL already treated in the frame of very intensive front-line and relapsed protocols.

**Abstract:**

Five-year event-free survival in pediatric B-cell precursor acute lymphoblastic leukemia (BCP-ALL) currently exceeds 80–85%. However, 15–20% of patients still experience a relapsed/refractory disease. From 1 January 2015 to 31 December 2020, thirty-nine patients, 0–21 years old with r/r BCP-ALL were treated with blinatumomab with the aim of inducing remission (*n* = 13) or reducing MRD levels (*n* = 26) in the frame of different multiagent chemotherapy schedules, in seven AIEOP centers. Patients were treated in compassionate and/or off-label settings and were not enrolled in any controlled clinical trials. Treatment was well tolerated; 22 (56.4%) patients reported adverse events (AE) on a total of 46 events registered, of which 27 (58.7%) were ≤2 grade according to CTCAE. Neurological AEs were 18 (39.1%); only two patients required transient blinatumomab discontinuation. Complete remission (CR) rate was 46% for the 13 patients treated with ≥5% blasts and 81% PCR/FC MRD negativity in the 26 patients with blasts < 5%. Median relapse-free survival was 33.4 months (95% CI; 7.5–59.3); median overall survival was not reached over a mean follow-up of 16 months. In our study, as in other real-life experiences, blinatumomab proved to be effective and well-tolerated, able to induce a high rate of CR and MRD negativity.

## 1. Introduction

Current risk-adapted protocols for children with B-cell precursor acute lymphoblastic leukemia (BCP-ALL) achieve 5-year overall survival (OS) rates exceeding 90% [1,2]. However, 15–20% of patients still experience a relapsed/refractory disease with 5-year OS in first relapse of about 50% [3,4,5]. Primary refractory disease and second or greater relapse have a dismal prognosis even with intensive chemotherapy treatment and allogeneic hematopoietic stem-cell transplantation (allo-HSCT). Antigen-targeted therapy approaches have significantly improved the prognosis of pediatric relapsed/refractory BCP-ALL (r/r BCP-ALL) over the past decade [6,7,8].

Blinatumomab is a bispecific T-cell engager (BiTE) with two different single-chain Fv fragments binding T-cell CD3 and B-cell CD19 antigens [9]. It has been fully approved since July 2017 by the Food and Drug Administration (FDA) and European Medicines Agency (EMA) for treatment of adults and children with r/r BCP-ALL; in March 2018, the FDA expanded approval to treat minimal residual disease [10,11,12,13,14]. The ALCANTARA single-arm, phase 2, multicenter trial, demonstrated blinatumomab response rate benefit in adult patients with Ph-positive disease [15].

We conducted a retrospective multicentric observational study (BLIN-COMPASS) to evaluate the real-life experience with blinatumomab in compassionate and off-label settings in pediatric/adolescent patients with r/r BCP-ALL with the aim of achieving CR or reducing the levels of MRD. The treatment was delivered in all cases after informed consent was provided by the parents or legal guardians and approved by local Institutional Review Boards (protocol code 29/20 of 15 April 2020).

## 2. Materials and Methods

Data of patients aged 0–21 years, with CD-19 positive r/r BCP-ALL, treated with blinatumomab in a compassionate or off-label setting and not enrolled in any additional experimental clinical trials, were collected between 1 January 2015 and 31 December 2020 from seven Italian pediatric hematology centers of Associazione Italiana di Ematologia e Oncologia Pediatrica (AIEOP).

The main goals of this study were to evaluate the toxicity as incidence of treatment-related adverse events (AEs) and the efficacy pattern as: (i) morphologic complete remission (CR) rate, defined as <5% blasts in bone marrow (BM) smears; (ii) minimal residual disease (MRD) response, defined as MRD level <1 × 10^−4^ by flow cytometry (FC) or polymerase-chain reaction (PCR) analysis; (iii) OS, defined as the time from the first blinatumomab administration and the last follow-up or death for any reason; and (iv) relapse-free survival (RFS), defined as time from blinatumomab infusion and date of relapse or death in remission, whichever occurred first.

All the analyses were performed using IBM SPSS Statistics, version 26; missing data were excluded. Survival analyses were conducted using the Kaplan–Meyer (KM) method, and time variables were defined in the aim of the study; for surviving patients, OS and RFS were censored at the date of last follow-up. For the survival analyses, patients were divided into two groups according to the bone marrow blast percentage at the time of blinatumomab administration: <5% and ≥5%. A one-sided stratified log-rank (Mantel–Cox) test was used to compare disease-free survival and overall survival between patient groups, with a significance threshold of one-sided *p* = 0.05. Fisher’s exact tests were used to examine the differences between categorical parameters, and statistical significance was set at *p* < 0.05.

## 3. Results

### 3.1. Study Population

A total of 39 patients were identified and met criteria for analysis. The median age was 9 years (range, 2–21 years) at the time of blinatumomab treatment. The disease was in first relapse in 23 (59%) and in second or later relapse in 12 patients (31%). Four (10%) patients, who had to discontinue first-line induction therapy for toxicity, showed persistent disease (2 with morphological blasts, 1% and 4%, respectively; 2 with PCR-MRD persistence, >5 × 10^−4^ and >1 × 10^−4^, respectively).

A total of 10 out of 39 patients (25%) had experienced relapse after HSCT, 4 of them relapsed after two allo-HSCT and one patient presented MRD-positivity after CAR-T cell therapy.

Seven subjects carried high-risk genetic abnormalities: four KMT2A rearrangement; one intra-chromosomal amplification of chromosome 21 (iAMP21); two Ph-like ALL.

At the time of blinatumomab administration, 26 patients (66.7%) had <5% BM blasts; 18 of them had previously received a debulking chemotherapy; 5 patients showed a molecular disease (PCR-MRD > 5 × 10^−4^); and 3 an FC-MRD positivity (blasts 1%, 2.9%, 4%). The other thirteen patients (33.3%) were referred to blinatumomab with an elevated leukemia burden (median blasts 30%; range 5–81%) (Table 1).

Blinatumomab was administered for a median of two courses (range 1–9); 17 patients received one cycle, eighteen 2 cycles and 4 underwent more than 2 courses. Most patients (21/39, 53%) received the pediatric dosage of stepwise escalation from 5 to 15 µg/m^2^/day in the first course and then 15 µg/m^2^/day in the subsequent courses; fourteen patients (36%) received the full pediatric dosage from the first day; four were treated with the adult schedule, three of them with the escalation from 9 to 28 µg/day and one with 28 µg/day from day one.

### 3.2. Safety Profile

AEs were graded according to the Common Terminology Criteria for Adverse Events (CTCAE v 4.03).

A total of 22 (56.4%) out of 39 patients experienced at least one treatment-related AE. A total of 46 AEs were reported, 27 (58.7%) of grade 1–2, 15 (32.6%) of grade 3, only 1 (2.2%) of grade 4 and 3 events (6.5%) of unknown grade.

There were 19 patients (48.7%) who experienced AEs in the first course; 4 out of 18 (22.2%) patients who received two therapeutic courses presented AEs in both courses; no AEs were registered in the third or fourth blinatumomab course (4 patients). Toxicity data were missing in three patients.

Fourteen patients (36%) experienced neurological events (7 ≤ grade 2); three patients with seizure were reported, and two required transient blinatumomab discontinuation. Two patients displayed tremor and two peripheral neuropathies. One patient reported aphasia and dysarthria associated with tremor and dysmetria (grade 3). Two patients had multiple neurological AEs, for a total recorded of 18 events.

One patient discontinued the first cycle because of multiple AEs: hematological toxicity (grade 3 anemia and thrombocytopenia), fever (grade 2), hypogammaglobulinemia (grade 2), progression of previous hepatic candidiasis and pneumocystis pneumonia. The same patient continued with the second course and developed liver toxicity with ascites (grade 2) and seizure (grade 3) due to severe hypokalemia.

The second most frequent AE was fever of unknown origin (11 patients), with grade 3 only in one case.

Hematological toxicity was the third most common adverse event (10.9%); only one case of combined anemia and thrombocytopenia grade 3 was registered; neutropenia of grade 2 was present in another patient.

No cytokine release syndrome (CRS) was reported. In our cohort of patients, the correlation between grade 3–4 severity of AEs and bone marrow infiltration ≥ 5% did not appear significant (Fisher’s exact tests one-sided *p* = 0.31). Type and frequencies of AEs distribution are shown in Table 2.

### 3.3. Treatment Outcomes

All 39 patients had detectable disease at the time of first blinatumomab course; 26 (66.6%) of them had <5% blasts, and 13 patients had overt disease with ≥5 % BM blasts.

A total of 23 (88.5%) out of the 26 patients with <5% of BM blasts at start of treatment sustained the CR; 19 (73%) of them achieved PCR-MRD negativity; 2 (7.7%) had FC-MRD negativity with unknown PCR-MRD status; 1 patient (3.8%) reached flow-cytometry negativity, but PCR-MRD persisted ≥ 10^−3^, and one (3.8%) was in morphological CR with persisted FC-MRD of 0.12%. Resuming, 81% of patients with <5% of blasts (21/26) obtained PCR/FC-MRD negativity.

In the 13 patients with BM blasts ≥ 5%, CR was obtained in 6 (46.1%); 5 (38.5%) of them achieved MRD negativity (2 by PCR and 3 by FC) and 1 patient (7.7%) had hematological CR, but showed FC-MRD positivity ≥ 10^−2^.

A total of 10 subjects (25.6%), 3 (11.5%) with <5% and 7 (54%) with ≥5% BM blasts, who did not achieve a response; 3 of them discontinued blinatumomab on day 11, 15 and 17 because of disease progression.

Twenty-nine patients (74%) obtained/sustained CR after the first blinatumomab course.

Three patients (10%) early relapsed after the second course, two after achieving a PCR-MRD negativity and one with persisted FC-MRD of 0.12% after the first cycle; one patient (3.4%) experienced a CD19 negative relapse during the third cycle and no other patients displayed a CD19 negative switch; consequently, the overall CR rate, after three courses, was 64%.

The response rate after the first blinatumomab course, assessed as FC/PCR MRD level < 1 × 10^−4^ for patients treated with <5% of BM blasts, and obtaining CR for those with overt disease, was significantly higher for patients treated with <5% of BM blasts compared with those with ≥5% (21 vs. 6; 95% CI *p* = 0.011); the difference was still significant after the third course (19 vs. 5; 95% CI *p* = 0.018), considering the cases of early relapse.

Among the four patients with KMT2A rearrangement: two were non-responders to blinatumomab, one obtained morphologic and FC-MRD response with PCR-MRD persistently ≥ 10^−2^, and only one achieved PCR-MRD negativity. The remaining three patients with high-risk genetic abnormalities showed complete PCR-MRD negativity after the first blinatumomab course.

A total of 21 patients proceeded to allo-HSCT during the follow-up; 16 without other bridging therapy (14 PCR-MRD negative; 1 FC-MRD negative and unknown MRD status and 1 PCR-MRD positive ≥ 10^−2^). Five patients, three non-responders and two relapsed, underwent HSCT after further chemotherapy. A total of 18 subjects never underwent HSCT—10 in CR, 7 non-responders and 1 relapsed after the second course.

A total of 11 of the 29 patients in CR after the first blinatumomab course relapsed (relapse rate 38%). Among the relapsed patients, three had reached MRD response (two FC-MRD and one PCR-MRD negativity), and one was in morphological CR with persistent FC-MRD of 0.12% after the first cycle. Four patients relapsed after HSCT; the remaining three patients relapsed one month, twelve months and thirty-three months after the end of the blinatumomab infusion, respectively.

Fourteen patients died during the observation period; nine of them never achieved remission (seven belonged to the group with ≥5% of BM blasts and two to the group with <5% of BM blasts at the time of blinatumomab administration); two had relapsed after the second course; one patient, had relapsed after ten months and died from sepsis; one patient had relapsed after 11 months, and one relapsed six months after HSCT.

**Figure 1:**
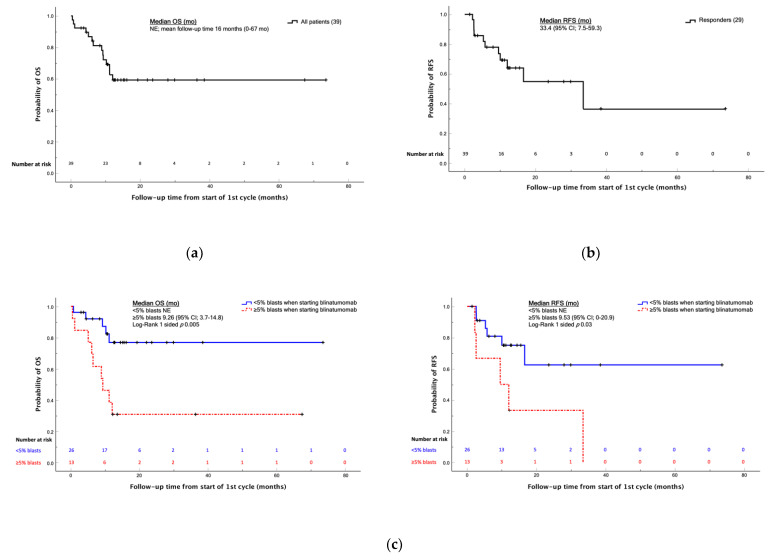
Kaplan-Meier analysis of the OS and EFS values in the cohort of patients between 2015 and 2020. (**a**) OS curve of the total cohort of 39 patients, median not evaluable over a mean follow-up of 16 months; (**b**) RFS curve of patients achieving remission, median 33.4 months; (**c**) OS and RFS curves of patients with <5% and ≥5% blasts at the time of blinatumomab administration. Abbreviations: OS, overall survival; RFS, relapse-free survival; mo, months; NE, not evaluable.

The median OS was not reached over a mean follow-up of 16 months (0–67 months) (Figure 1a); no difference was found in median OS between patients in first and second or greater relapse (*p* = 0.10). Median RFS for a patient who achieved remission was 33.4 months (95%CI; 7.5–59.3) (Figure 1b).

**Figure 2:**
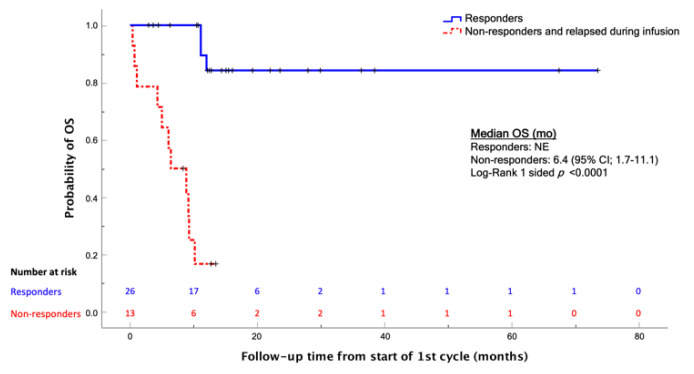
Kaplan-Meier analysis of the OS comparing patients who achieved CR after blinatumomab, and those who did not; log-Rank one-sided *p* < 0.0001. Abbreviations: OS, overall survival; mo, months; NE, not evaluable.

As expected, OS was statistically better for patients who achieved a CR compared with those who did not (*p* < 0.0001) (Figure 2).

The cohort of patients with <5% of blasts showed a better RFS and OS than those with ≥5% (*p* = 0.03 and *p* = 0.005, respectively) (Figure 1c).

**Figure 3:**
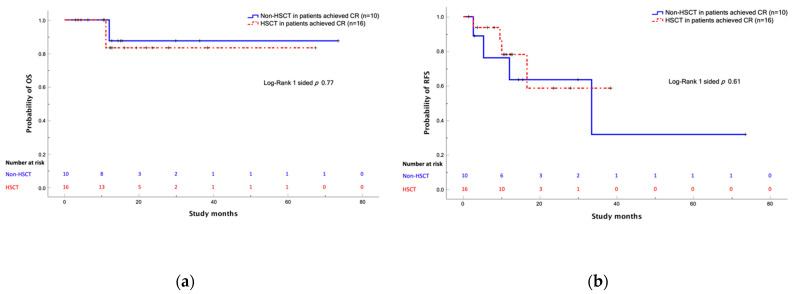
Comparison between patients who underwent HSCT and those who did not after achieving CR with blinatumomab treatment. (**a**) OS curves; (**b**) RFS curves. Abbreviations: OS, overall survival; HSCT, hematopoietic stem cell transplantation; CR, complete remission.

**Figure 4:**
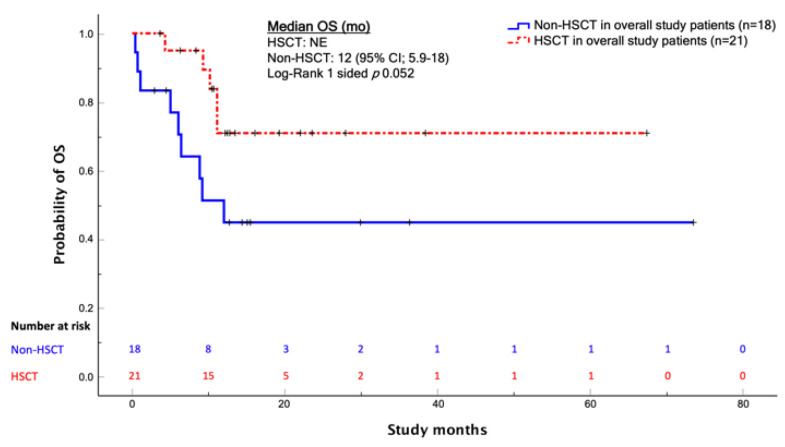
Kaplan-Meier analysis of the OS comparing patients who underwent HSCT and those who did not. The difference was not significant; log-rank one-sided *p* = 0.052. Abbreviations: OS, overall survival; mo, months; HSCT, hematopoietic stem cell transplantation; NE, not evaluable.

Among patients in CR after blinatumomab therapy, there was no evidence that HSCT had a survival benefit in terms of OS or RFS (*p* = 0.77 and 0.61, respectively) (Figure 3); however, among the overall study patients, HSCT showed an OS benefit, though not statistically significant (*p* = 0.052) (Figure 4).

## 4. Discussion

This retrospective analysis is one of the largest multicenter real-life studies on r/r BCP-ALL pediatric patients treated with blinatumomab in compassionate or off-label settings. Unlike the recent German experience [16], reporting a similar sample size, we did not include data from patients enrolled in controlled clinical trials.

The encouraging results obtained with blinatumomab in adult patients with ALL [12,15] led to an increasingly progressive use also in the pediatric population, where ALL is the most common malignant disease with about 15% of relapses and 10% of disease-related deaths [17]. Furthermore, early- and long-term intensive chemotherapy-related toxicities are a serious issue for pediatric patients with a long-life expectancy. In this scenario, blinatumomab proved to be a new therapeutic model of great efficacy with a good safety profile [18,19,20,21,22,23].

In recent years, different retrospective studies have evaluated blinatumomab efficacy and safety in pediatric r/r BCP-ALL within compassionate or off-label use outside of any clinical trial program.

Queudeville et al. [16] reported 38 patients treated with blinatumomab over 10 years—71% with a bone marrow infiltration above 25%. Toxicity was noted in this study; seven patients (18%) presented neurotoxicity, and two discontinued therapy due to generalized seizures; about half of the patients (20/38, 52%) experienced a CRS, with a clear association between leukemia burden and the CRS level; hematological toxicity was reported in 63% of patients suffering from grade 3 and 4 anemia and/or thrombocytopenia and in 83% of patients that presented grade 3 and 4 neutropenia.

Comparable data were reported by Contreras et al. [24]—neurological complications accounted for 22% (4/18 patients), of which only one was grade 4, while a lower percentage of CRS was described (6/18 patients, 33%).

The SHEOP group [25] described 15 patients with a very low number of non-hematological AEs: one patient with grade 3 neurotoxicity (dysarthria, paresthesia and somnolence) recovered after blinatumomab discontinuation, as did another with grade 4 hypokalemia and two subjects with grade 3 hepatobiliary disorder; in contrast, grade 3/4 hematological toxicity was reported in 11 of 15 treated patients.

Similarly, Ampatzidou et al. [26] described nine patients with a low toxicity profile; three patient developed neurotoxicity, with two of them requiring transient discontinuation, but no CRS was reported.

In our analysis we observed a major number of neurological events (14/39 patients, 36%), mostly of grade ≤ 3, and two patients with seizure who needed transient blinatumomab discontinuation. In contrast, a lower rate of hematological toxicities and no CRS were reported. Probably the higher number of patients with <5% blasts recruited in our cohort could explain the emerged differences, supported by the hypothesis that a low leukemic burden is related to an off-target effect of blinatumomab due to non-specific T cell activation [27].

Although limited by the sample size, the heterogeneity of cohorts and of previous treatments and different methodology of response evaluation, a remarkable agreement has emerged from data obtained in real-life experiences recently published, underlining the importance of these retrospective studies in providing valuable safety and efficacy information. Clearly, the results of these studies are difficult to compare in terms of efficacy due to the relevant differences in the populations treated; however, blinatumomab appears capable of inducing CR in a percentage of patients ranging between 34% [16] and 66.7% [26]. In the German [16] retrospective analysis, the median OS for all patients was 11.1 months (0.2–113 months), and median RFS was 6.17 months (0–18 months), with a total CR rate of 34%. Contreras et al. [24] reported 18 patients treated with commercial blinatumomab; 12 of them achieved a CR (both morphological and MRD). In the Spanish study [25] 8 of 15 children (53%) reached CR with sustained MRD negativity after one cycle; median OS and RFS were 22 (range 3–41) and 8.5 months (range 0–17), respectively. Ampatzidou et al. [26] reported 66.7% of CR after the first cycle in nine patients but a considerably lower OS (8.7 months, 1.4–47.1) and RFS (3 months, 0.5–21.4). Recently, the Australian group [28] published their experience with blinatumomab in 24 high-risk genetics r/r BCP-ALL patients, showing 58% of MRD response rate. However, patients with KMT2A rearrangements had poor outcomes, with MRD response rate of 44%, 2-year progression-free survival (PFS) of 15% and 2-year OS of 37%.

Compared with these studies, our case series showed in patients with <5% blasts at baseline (66%) a very good response in terms of MRD (81%) and, consequently, better OS and RFS rates.

With regard to KMT2A rearranged patients and as in other reports [28], our analyses showed a response rate of 50% and a 2-year OS and 2-year PFS of 50% and 25%, respectively; however, the very small number of our patients does not allow definitive conclusions.

Our study emphasized controversial results about the role of HSCT in blinatumomab responders vs. non-responders. In fact, we did not observe a survival benefit in terms of OS or RFS (*p* = 0.77 and 0.61, respectively) in transplanted patients with respect to patients not transplanted, but this is most probably related to the small numbers and to the relatively brief observation period. However, a slight OS improvement in transplanted patients emerged when analyzing the total cohort of patients (*p* = 0.052); our results agree with the experience of Jabbour in the phase 3 study on adult patients [29]. In contrast, Queudeville et al. [16] demonstrated a positive effect on OS of HSCT after blinatumomab, while the RIALTO trial [23] showed only a trend towards improving OS and RFS.

The efficacy of blinatumomab, already demonstrated in the phase 1/2 trial in heavily pretreated pediatric r/r BCP-ALL [22,23], was recently compared with the standard of care (SOC) [30] of three historical groups from North America, Australia and Europe, resulting in higher CR and longer OS rates.

Currently blinatumomab has also been successfully used in first relapse BCP-ALL. Brown and colleagues [31] described the effect of consolidation therapy with blinatumomab, reporting a better 2-year DFS (54.4% vs. 39%, *p* = 0.03) and OS (71.3% vs. 58.4%, *p* = 0.02), a higher rate of MRD negativity (75% vs. 32%, *p* < 0.001) and a lower rate of adverse events. A total of 70% of patients proceeded to HSCT, compared with 43% in the control group (*p* < 0.001). Locatelli et al. [32] evaluated the role of blinatumomab in reducing residual leukemia burden before HSCT in high-risk first-relapse BCP-ALL, observing an inferior incidence of events (31% vs. 57%, *p* < 0.001), a superior rate of MRD remission (90% vs. 54%) and improved EFS (22.4-month IQR 8.1–34.2). Regarding toxicities, fewer adverse events were observed in the blinatumomab arm vs. conventional chemotherapy, particularly for hematological toxicities such as febrile neutropenia [31,32]. Brown et al. described 22% CRS, 11% encephalopathy and 4% seizures, but all of these AEs were fully reversible, and there were no AE-related deaths [31]. Locatelli et al. reported only three cases of grade 3 or 4 neurologic events and no CRS grade ≥ 3 [32].

Considering the good short-term toxicity profile that emerged in our retrospective experience, and even with the clear limitations of the retrospective nature of these evaluations (case-series), our data confirm that molecular and morphological CR can be obtained in a relevant percentage of r/r BCP ALL children and add further elements to the limited real-life experience of the use of blinatumomab that is already available in this setting. Ongoing international observational retrospective studies (NEUF trial) [33] could provide additional information about long-term toxicities and efficacy in the pediatric real-life setting. Blinatumomab is currently under clinical investigation in front-line treatment for children with standard-risk ALL (NCT03914625, NCT02877303), for minimal residual disease to the end of induction therapy (NCT03117751) and in intermediate or high-risk ALL (NCT0363276) [20].

## 5. Conclusions

Blinatumomab has changed the treatment paradigm of r/r BCP-ALL in second or greater relapse, not only because of its efficacy but also because of its toxicity profile, and it also seems to give better chances of undergoing HSCT in deep MRD remission and in better performance status. Further studies are needed to ascertain the role of blinatumomab in a setting that is HSCT-free or even whether it has any role post-transplantation, in order to establish the best timing and setting to locate its use in childhood ALL treatment.

## Figures and Tables

**Table 1:** Characteristics of the 39 patients enrolled in the observational study.

Characteristics	*n* (%)
Total Patients	39
Sex	
Male	22 (56.4)
Female	17 (43.6)
Age at initial diagnosis	
Median, years (IQR)	5.3 (0.2–20.4)
<12 months	3 (7.7)
1–18 years	35 (89.7)
>18 years	1 (2.6)
Immunophenotype	
pre-B (EGIL B-I)	8 (20.5)
Common (EGIL B-II)	27 (69.2)
pro-B (EGIL B-III)	4 (10.3)
Karyotype	
Hyperdiploid	7 (17.9)
t (12; 21)	1 (2.6)
t (3; 9)	1 (2.6)
Other chromosomal aberrations	3 (7.7)
Normal karyotype	9 (23.1)
Unknown cytogenetic status	18 (46.1)
Molecular abnormalities	
iAMP21	1 (2.6)
Ph-like	2 (5.1)
KMT2A-rearrangement	4 (10.3)
Absence of mutations	24 (61.5)
Unknown mutational status	8 (20.5)
First-line risk stratification	
Standard Risk	4 (10.3)
Medium Risk	13 (33.3)
High risk	15 (38.5)
Unknown	7 (17.9)
Months from diagnosis to blinatumomab treatment	
Median (IQR)	6 (0.7–20.1)
<18 months	9 (23.1)
18 months–30 months	8 (20.5)
>30 months	22 (56.4)
Age at treatment	
Median, years (IQR)	9.2 (2.3–21.5)
<12 months	0 (0)
1–18 years	36 (92.3)
>18 years	3 (7.7)
Therapeutic line	
Front line	4 (10.2)
First relapse	23 (59)
Second or greater relapse	12 (30.8)
Refractory	4 (10.2)
Relapses	35 (89.7)
Site of relapse	
Bone Marrow	24(68.5)
Molecular relapse	3(8.6)
Combined relapse	8 (22.9)
HSCT pre blinatumomab	
Yes	10 (25.6)
No	29 (74.4)
Blinatumomab courses	
1	17 (43.6)
2	18 (46.2)
>2	4 (10.2)
Bone marrow blasts before blinatumomab <5%	26 (66.7)
After debulking chemotherapy	18 (46.2)
PCR-MRD positive	5 (12.8)
FC-MRD positive	3 (7.7)
Bone marrow blasts before blinatumomab ≥5%	13 (33.3)
>5–25% blasts	6 (15.4)
>25% blasts	7 (17.9)

IQR: Interquartile Range; EGIL: European Group for the Immunological Characterization of Leukemias; iAMP21: intrachromosomal amplification of chromosome 21; HSCT: Hematopoietic Stem Cell Transplantation.

**Table 2:** Adverse events registered according to the Common Terminology Criteria for Adverse Events (CTCAE v 4.03), number and percentage for each event and grading.

Toxicities During Blinatumomab	Grade 1–2		Grade 3		Grade 4		Unknown Grade		Total	
	*n*	%	*n*	%	*n*	%	*n*	%	*n*	%
Patients with at least one adverse event									22	56.40%
Total adverse events									46	100%
Neurotoxicity	7	15.2%	9	19.6%	1	2.2%	1	2.2%	18	39.1%
Unspecified neurotoxicity	3	6.5%	2	4.3%	-	-	-	-	5	10.9%
Altered mental status	1	2.2%	1	2.2%	-	-	-	-	2	4.3%
Dysmetria	-	-	1	2.2%	-	-	-	-	1	2.2%
Aphasia	-	-	1	2.2%	-	-	-	-	1	2.2%
Dysarthria	-	-	1	2.2%	-	-	-	-	1	2.2%
Seizure	1	2.2%	1	2.2%	1	2.2%	-	-	3	6.5%
Tremor	1	2.2%	1	2.2%	-	-	1	2.2%	3	6.5%
Polyneuropathy	1	2.2%	1	2.2%	-	-	-	-	2	4.3%
Hepatotoxicity	3	6.5%	1	2.2%	-	-	-	-	4	8.7%
Fever	8	17.4%	1	2.2%	-	-	2	4.3%	11	24%
Rash	2	4.3%	-	-	-	-	-	-	2	4.3%
Hematological toxicity	3	6.5%	2	4.3%	-	-	-	-	5	10.9%
Thrombocytopenia	1	2.2%	1	2.2%	-	-	-	-	2	4.3%
Anemia	1	2.2%	1	2.2%	-	-	-	-	2	4.3%
Neutropenia	1	2.2%	-	-	-	-	-	-	1	2.2%
Progressive candidiasis	-	-	1	2.2%	-	-	-	-	1	2.2%
Pneumocystosis	-	-	1	2.2%	-	-	-	-	1	2.2%
Ascites	1	2.2%	-	-	-	-	-	-	1	2.2%
Hypogammaglobulinemia	2	4.3%	-	-	-	-	-	-	2	4.3%
Autoimmune arthritis	1	2.2%	-	-	-	-	-	-	1	2.2%

## Data Availability

The data presented in this study are available on request from the corresponding author.
